# X-Linked Alport Dogs Demonstrate Mesangial Filopodial Invasion of the Capillary Tuft as an Early Event in Glomerular Damage

**DOI:** 10.1371/journal.pone.0168343

**Published:** 2016-12-13

**Authors:** Sabrina D. Clark, Mary B. Nabity, Rachel E. Cianciolo, Brianna Dufek, Dominic Cosgrove

**Affiliations:** 1 Department of Veterinary Pathobiology, Texas A&M University College of Veterinary Medicine & Biomedical Sciences, College Station, Texas, United States of America; 2 Department of Veterinary Biosciences, The Ohio State University Columbus, Ohio, United States of America; 3 Center for Basic Research, Boys Town National Research Hospital, Omaha, Nebraska, United States of America; Cedars-Sinai Medical Center, UNITED STATES

## Abstract

**Background:**

X-linked Alport syndrome (XLAS), caused by mutations in the type IV collagen COL4A5 gene, accounts for approximately 80% of human Alport syndrome. Dogs with XLAS have a similar clinical progression. Prior studies in autosomal recessive Alport mice demonstrated early mesangial cell invasion as the source of laminin 211 in the glomerular basement membrane (GBM), leading to proinflammatory signaling. The objective of this study was to verify this process in XLAS dogs.

**Methods:**

XLAS dogs and WT littermates were monitored with serial clinicopathologic data and kidney biopsies. Biopsies were obtained at set milestones defined by the onset of microalbuminuria (MA), overt proteinuria, onset of azotemia, moderate azotemia, and euthanasia. Kidney biopsies were analyzed by histopathology, immunohistochemistry, and electron microscopy.

**Results:**

XLAS dogs showed progressive decrease in renal function and progressive increase in interstitial fibrosis and glomerulosclerosis (based on light microscopy and immunostaining for fibronectin). The only identifiable structural abnormality at the time of microalbuminuria was ultrastructural evidence of mild segmental GBM multilamination, which was more extensive when overt proteinuria developed. Co-localization studies showed that mesangial laminin 211 and integrin α8β1 accumulated in the GBM at the onset of overt proteinuria and coincided with ultrastructural evidence of mild cellular interpositioning, consistent with invasion of the capillary loops by mesangial cell processes.

**Conclusion:**

In a large animal model, the induction of mesangial filopodial invasion of the glomerular capillary loop leading to the irregular deposition of laminin 211 is an early initiating event in Alport glomerular pathology.

## Introduction

The glomerular basement membrane (GBM) is extracellular matrix that is situated between podocytes and endothelium and is composed of a meshwork of type IV collagen, laminin 521, nidogen, and the heparan sulfate proteoglycan agrin. The GBM plays an integral role in glomerular filtration through both size and charge-selectivity.[1, 2] Additionally, the GBM deviates from its pericapillary course and extends out to cover the base of the capillary loop between the mesangial cells and the podocytes.[3]

Approximately 50% of the GBM is composed of type IV collagen, which is essential in maintaining both stability and function.[4] Type IV collagen alpha chains form heterotrimers which self-assemble forming a tissue-specific cross-linked network.[1, 4] During nephrogenesis, the GBM is composed exclusively of α1α1α2 type IV collagen. As the glomerulus matures, the sub-epithelial α1α1α2 network is replaced by α3α4α5 type IV collagen secreted by the podocytes, which predominates in the mature GBM.[5, 6] The α3α4α5 type IV collagen network is more heavily cross-linked and protease-resistant than α1α1α2, and is therefore better suited for maintaining GBM integrity from increasing hydrostatic pressure to which glomeruli are exposed.[2, 7–9] Laminin is the most prevalent non-collagenous protein of the GBM. These cross-shaped heterotrimers consists of an α, β, and γ chain with sixteen different isoforms being identified.[10] The mature GBM is comprised of laminin 521 (α5β2γ1).

Alport Syndrome (AS) is a hereditary disease that has been characterized in mice, dogs, and humans.[11–16] It is caused by mutations in the α3, α4, or α5 type IV collagen genes, primarily leading to delayed-onset progressive glomerulopathy. While mice and dogs tend to develop proteinuria first compared to humans who present with hematuria, the disease has similar renal clinical manifestations in all species, eventually end-stage renal disease. Additionally, affected human males will often manifest extra-renal disease, including sensorineural deafness and ocular abnormalities.[17] While aural and ocular abnormalities have been identified in mice, thorough evaluation of these systems has not yet been performed in dogs. It has been noted, however, that dogs do not exhibit signs that indicate these abnormalities are present.[14, 16, 18] Two main forms of AS exist. X-linked AS (XLAS) is due to a mutation in the COL4A5 gene and accounts for approximately 80% of cases. Autosomal AS (dominant or recessive) results from a mutation in either COL4A3 or COL4A4 and accounts for the remainder of the cases.[12] Because of the way collagen is assembled, a mutation in any one of the α chain genes prevents proper formation of the α3α4α5 type IV collagen protomer resulting in absence of the sub-epithelial α3α4α5 type IV collagen network and a GBM comprised only of α1α1α2 type IV collagen. With fewer interchain crosslinks, this change in composition compromises the long term integrity of the GBM.[2, 19, 20] On renal biopsy, the loss of α3α4α5 results in thinning and thickening of the GBM often referred to as a “basket weave” appearance on electron microscopy that is pathognomonic for the disease and serves as a definitive diagnostic test.[7, 12, 17, 19]

In normal glomeruli, laminin 211 (α2β1γ1) is located within the mesangium but not the GBM. Evaluation of glomeruli obtained from mice, dogs, and humans in the early stages of AS show a distinctive feature of aberrant laminin deposits within the GBM, including patchy, non-linear deposits of laminin 211, regardless of the mode of inheritance.[21] However, the source of this abnormal deposition was unknown.[21, 22] Using integrin α1-deficient mice crossed with AS mice to create a double knockout, the abnormal deposits of laminin 211 were implicated in the degradation of the GBM early in the disease process.[22] In autosomal recessive 129/Sv Alport mice, GBM laminin 211 was shown to originate from mesangial cell filopodia that progressively invaded capillary loops.[23] Additionally, biomechanical strain in the capillary wall due to the thinner GBM and fewer cross-links of α1α1α2 type IV collagen is associated with induction of mesangial cell process invasion, contributing to initiation and progression of disease.[23] Furthermore, focal adhesion kinase (FAK) activation occurs specifically in regions where abnormal laminin is present, causing increased expression of interleukin-6 (IL-6) and matrix metalloproteinases (MMPs), particularly MMP-9, MMP-10, and MMP-12, all of which contribute to disease progression by propagating GBM destruction.[24–27]

Advancement in understanding the molecular mechanism of AS progression has been established primarily using murine models. While mice have rapid progression of disease and are less expensive compared to large animal models, they also possess a number of limitations.[28] They lack genetic heterogeneity, have different immune and metabolic responses, and knockout mouse models do not always emulate human disease.[29, 30] Large animal models provide a strong link from mice to humans, particularly for testing of therapeutic efficacy. Thus identification of these models is imperative. Here, we demonstrate that the mechanism of pathogenesis of canine XLAS is similar to that of mice, thereby supporting that the pathogenesis in humans is also likely comparable. Additionally, we provide evidence that the dog is a suitable large animal model for evaluation of AS progression and novel therapeutic trials.

## Materials and Methods

### Animals

Dogs were from a colony maintained at Texas A&M University, in which the causative mutation of the disease in the affected (AS) males was a naturally occurring 10 base pair deletion in the gene encoding the α5 chain of type IV collagen.[13] Development and progression of X-linked hereditary nephropathy (XLHN) in these dogs has been described.[31] Analysis of physiologic and histopathologic data was performed on eight adolescent male dogs with AS and four unaffected, wild type (WT), age-matched male littermates. Immunostaining was performed on two AS and two WT male dogs. No treatments were administered to the dogs used in the study. The study protocol was reviewed and approved by the Texas A&M University Institutional Animal Care and Use Committee. Dogs for this study were raised using a standardized protocol that included housing in temperature-regulated, indoor, individual runs with a 12-hour light-dark cycle. Depending on weather conditions, dogs were provided daily leash-walks outside or received individual or group unrestricted playtime in an outdoor grass pen with access to a variety of toys and/or a wading pool during the spring and summer months. Dogs were fed once daily in the morning after urine collection. Their diets consisted of a mix of Purina ProPlan Focus Puppy canned & dry dog food until study entry at around seven weeks of age. They were then placed on Purina ProPlan Savor Chicken & Rice Classic Adult canned food for the remainder of the study period.

### Sample Collection

Starting at 7 weeks of age, blood and mid-stream voided urine were collected on a weekly basis for evaluation. Physiologic data, including serum creatinine (sCr), urine protein: urine creatinine ratio (UPC), and symmetric dimethylarginine (SDMA) were used as previously described [32] to detect advancement of disease defined by set milestones (MS). SDMA is a methylated arginine that is released in the blood during protein degradation and excreted by the kidneys. In dogs, it serves as a useful marker for evaluation of decreasing renal function.[32] Milestones were defined by the following criteria: MS 1-presence of microalbuminuria for two consecutive weeks (average age 14.1 weeks), MS 2-UPC ≥ 2 for two consecutive weeks (average age 20.1 weeks), MS 3-sCr ≥ 1.2 mg/dL (average age 28.8 weeks), MS 4-sCr ≥ 2.4 (average age 35.0 weeks), and MS 5-sCr ≥ 5 mg/dL (average age 39.5 weeks). Testing for microalbuminuria was performed only until MS 1 was reached using a semi-quantitative test (E.R.D. HealthScreen Canine Urine Test strips, Heska, Loveland, Colorado). Glomerular filtration rate was determined by iohexol clearance using an 8-point sampling protocol starting at 9 weeks of age. GFR was determined monthly and when dogs reached each milestones of disease as described above. Additionally, ultrasound-guided needle biopsies of the kidneys (described below) were obtained from each dog when a defined MS was reached. WT dogs were paired with an AS littermate for milestone evaluation to serve as a control and were evaluated and biopsied at the same time as their AS counterpart.

For kidney biopsy collection, dogs were anesthetized using a premedication combination of 0.011 mg/kg glycopyrrolate (Fort Dodge, Overland Park, KS) and 0.30 mg/kg butorphanol (Zoetis, Florham Park, New Jersey) injected subcutaneously. Dogs were intubated following administration of 4–6 mg/kg propofol (Abbott, Worchester, Massachusetts) intravenously, and anesthesia was maintained using isoflurane (Zoetis Florham Park, New Jersey). Once the dog was fully anesthetized, biopsies were obtained using a 16–18 gauge Bard Monopty^®^ Disposable Core biopsy instrument. Biopsies were performed on alternating kidneys as each MS was reached. Samples were divided and placed into formalin, glutaraldehyde or Optimal Cutting Temperature (OCT) compound (Tissue-Tek OCT Compound, Sakura Finetek USA, Torrance, CA). Samples in OCT were flash frozen in liquid nitrogen vapor and stored at -80°C until evaluation. Dogs were not administered additional medication following the biopsy procedure as the butorphanol given during the pre-medication phase provided sufficient post-operative pain management. When AS dogs reached MS 5 or had clinically significant disease (i.e. an abrupt increase in sCr >5mg/dL and/or severe uremic signs related to chronic kidney disease (CKD)), they were humanely euthanized following biopsy collection. One of the affected dogs was euthanized prior to reaching MS 4 due to non-renal related disease. Euthanasia was performed while dogs were still under anesthesia by intravenous administration of a pentobarbital sodium solution (Fatal Plus, Vortech Pharmaceuticals, Ltd., Dearborn, Michigan).

### Light and Electron Microscopy Evaluation

For light microscopy, formalin-fixed, paraffin-embedded biopsies were sectioned at 3 μm and stained with H&E, Masson’s trichrome, and Periodic acid-Schiff. Sections were scored as previously described.[33] An average glomerulosclerosis score was determined for each milestone in both the WT and AS dogs using the following features: segmental sclerosis, global sclerosis, and synechiae. Similarly, an average tubulointerstitial damage score was determined using the following features: tubular dilation, loss of brush border, tubular atrophy, tubular epithelial cell degeneration/regeneration, tubular single cell necrosis, interstitial fibrosis, and chronic interstitial inflammation (nephritis).

For transmission electron microscopy (TEM), tissues were fixed in chilled 3% glutaraldehyde and post-fixed in 1% osmium tetroxide. Dehydration was performed using a series of alcohols followed by placement in an acetone/epoxy plastic for embedding. Semi-thin sections were cut with an ultramicrotome (EM UC6, Leica Microsystems, Buffalo Grove, IL) and stained with a mixture of Azure II and methylene blue. When the optimal area for evaluation was identified, ultrathin sections were cut (65–85 nm) and mounted on copper grids. The sections were post-stained with uranyl acetate and lead citrate. Grids were imaged on a JEOL JEM-1400 TEM (JEOL USA, Inc., Peabody MA) and photographed with an Olympus SIS Veleta 2K camera (Olympus Soft Imaging Solutions GmbH, Munster, Germany).

### Immunofluorescence Antibodies

The following antibodies were used: rabbit anti-mouse fibronectin (Sigma, St. Loius, MO, USA, Cat# F3648), goat anti-mouse integrin α8 (R&D Systems, Minneapolis, MN, USA, Cat# AF4076), mouse anti-bovine laminin β2 C4 (Developmental Studies Hybridoma Bank, University of Iowa, Iowa City, IA, USA), rabbit anti-mouse collagen IVα5 (Cosgrove) and rabbit anti-human laminin α2 (gift from Dr. Peter Yurchenco, Robert Wood Johnson Medical School, Piscataway, NJ, USA). Alexa-fluor conjugated secondary antibodies (Invitrogen, Carlsbad, CA, USA) included: donkey anti-rabbit 594 for anti-fibronectin and anti-collagen IVα5, and donkey anti-goat 568 for anti-integrin α8 and donkey anti-rabbit 488 for anti-laminin α2 or donkey anti-mouse 488 for anti-laminin β2 C4 (for dual staining). Negative controls were performed using the host serum in combination with the specific antibodies described above (S1 Fig).

### Immunofluorescence and Confocal Microscopy

Frozen OCT-embedded kidney biopsy samples were sectioned at 6 μm and acetone fixed. Sections were incubated overnight at 4°C in a primary antibody solution comprised of 0.3% PBST (Triton X-100), 5% fetal bovine serum, and the following antibody dilutions: 1:500 (fibronectin and collagen IVα5), 1:200 (integrin α8 and laminin α2) or 1:50 (laminin β2 C4). Slides were rinsed with 1X PBS and incubated at room temperature for 1 hour with the appropriate secondary antibody solution consisting of the secondary antibody along with 0.3% PBST (Triton X-100), and 5% fetal bovine serum to make a 1:500 antibody dilution. Slides were rinsed again with 1X PBS and mounted using Vectashield mounting medium, which contained DAPI to counterstain the nuclei (Vector, Burlingame, CA). Confocal images were captured using a Leica TCS SP8 MP microscope interfaced with a LSM510 META confocal imaging system, using either a 10x0.3 n.a. dry, 40x1.3 n.a. oil, or 63x1.4 n.a. oil objective (Carl Zeiss, Oberkochen, Germany). Final figures were assembled using Adobe Photoshop and Illustrator software (Adobe Systems, San Jose, CA).

### Statistical Analysis

Using JMP Pro 11.0, a Shapiro-Wilks Goodness of Fit test was performed on the residuals of sCr, SDMA, UPC, and iohexol clearance values along with glomerulosclerosis and tubulointerstitial fibrosis scores to determine normality. A Mann-Whitney U test was performed to determine statistical significance of clinicopathological data and light microscopy scores between WT and AS dogs at each milestone defined by a p-value of <0.05.

## Results

### Clinical Course of Dogs

**Fig 1:**
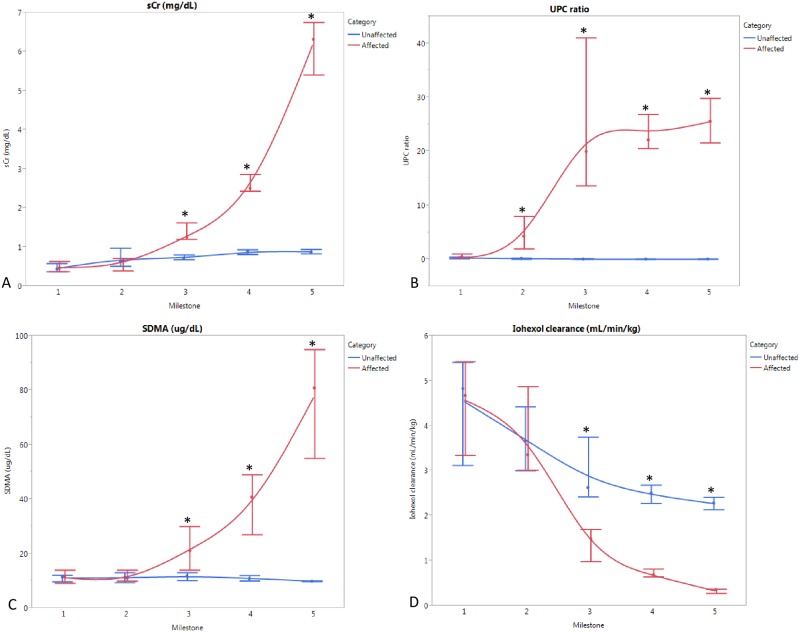
Clinical parameters (average, range) at each milestone in AS (n = 8) vs WT (n = 4) dogs. (A) Serum creatinine (sCr); (B) Symmetric dimethylarginine (SDMA); (C) Urine protein: urine creatinine (UPC); (D) Iohexol clearance; *p<0.05.

The first clinical indication of disease in AS dogs was the onset of microalbuminuria (MS 1) between 10–19 weeks of age (versus hematuria as the first detectable abnormality typically identified in humans). This progressively worsened to overt proteinuria (MS 2) between 14–29 weeks of age, followed by rapid advancement to renal failure. Between 26–52 weeks of age, AS dogs were euthanized following biopsy collections at end point (MS 5). Fig 1 summarizes the average values of clinicopathologic parameters at defined milestones for AS dogs compared with WT, age-matched littermates.

Estimates of GFR (sCr, SDMA, and iohexol clearance), which are commonly used to detect renal insufficiency, were not significantly altered until MS 3. Proteinuria based on UPC was significantly increased at MS 2 (Fig 1), and presence of microalbuminuria was the defining feature of MS 1. Therefore, proteinuria is more sensitive than GFR for identification of early events in disease development in dogs.

### Pathologic Evaluation

**Fig 2:**
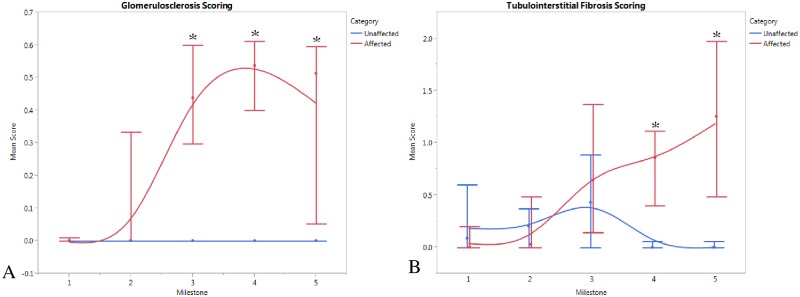
Pathologic parameters (average, range) at each milestone in AS (n = 8) vs WT (n = 4) dogs. (A) Glomerulosclerosis score; (B) Tubulointerstitial damage score; *p<0.05.

Light microscopy was also insensitive to early disease. Glomerulosclerosis and tubulointerstial fibrosis were not significantly increased until MS 3 and MS 4, respectively, corresponding with significant changes in the clinical estimates of GFR (Fig 2).

**Fig 3:**
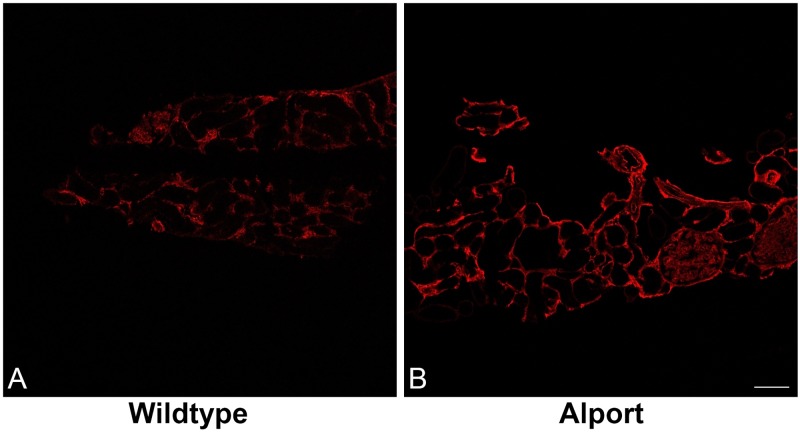
Immunofluorescence staining for fibronectin of kidney from WT and AS dogs at milestone 1. Staining for fibronectin reveals fibrosis in AS dogs (B) as early as milestone 1 on confocal microscopy when compared to WT littermates (A) at the same milestone, 10x0.3 n.a. dry.

However, on TEM of tissue from two AS dogs, mild, focal, segmental multilamination of the GBM was observed at MS1 (not shown). Additionally at MS 1, immunofluorescence showed increased staining for fibronectin both within the glomeruli and throughout the interstitium of AS dogs compared to WT dogs (Fig 3A and 3B), indicating initiation of fibrosis as early as the onset of microalbuminuria. Staining for fibronectin intensified with progression of disease at each MS (not shown).

### Detection of Mesangial Cell Invasion

**Fig 4:**
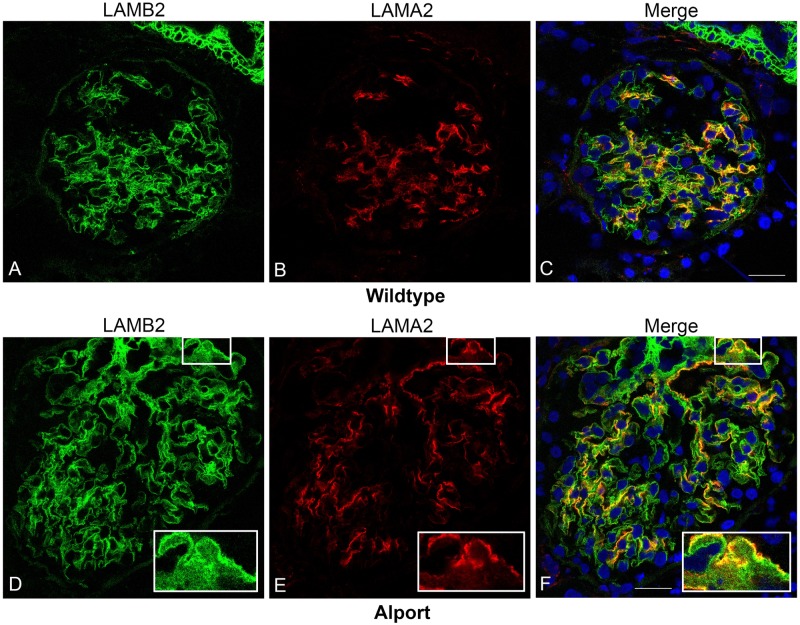
Identification of laminin 211 in the GBM of AS but not WT dogs. Dual immunofluorescence immunostaining of kidney from a WT dog (A-C) and an AS dog at milestone 2 (D-F); 63x1.4 n.a. oil. Laminin 521 of the GBM was labeled with anti-laminin β2 (LAMB2), and laminin 211 produced by mesangial cells, was labeled with anti-laminin α2 (LAMA2), demonstrating co-localization of laminin 211 with the GBM of several capillary loops in the AS dog.

The laminin β2 chain of laminin 521 is located in the GBM of mice, dogs, and humans[21] and thus can be used as a marker for the GBM in both WT and AS kidney tissue. In the normal glomerulus, laminin 211, identified by the laminin α2 chain, is found primarily within the mesangium and there is no expression of laminin 211 within the GBM of non-diseased kidney.[21] Fig 4A–4C, demonstrates the distinctness of laminin β2 as a GBM marker in normal canine kidney tissue and shows the more diffuse distribution of laminin α2 staining within the mesangium. In contrast, in the AS dog, there is segmental expression of abnormal deposits of laminin α2 in the GBM (Fig 4D–4F), particularly where the GBM is thickened.

**Fig 5:**
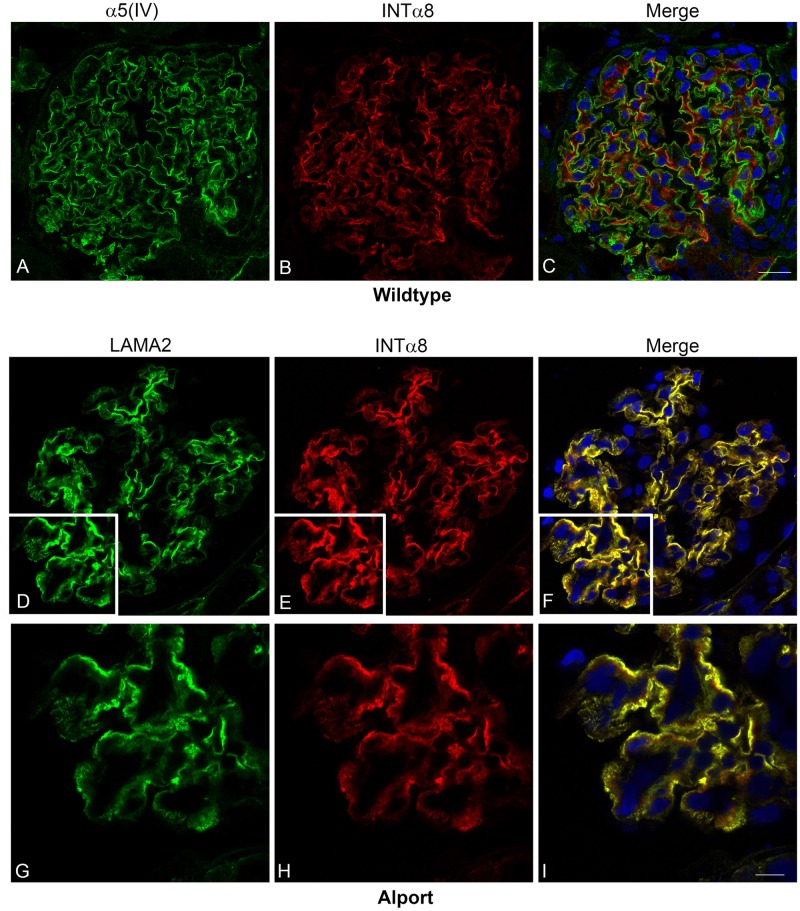
Integrin α8 co-localizes with laminin 211 in the GBM of AS but not WT dogs. A-C: Dual immunofluorescence immunostaining of kidney from a WT dog; 63x 1.4 n.a. oil. The GBM was localized with anti-collagen α5 (α5(IV)) and the mesangium was localized with anti-integrin α8 (INTα8). D-I: Dual immunofluorescence immunostaining of kidney tissue from an AS dog at milestone 2. Laminin 211, produced by mesangial cells, was labeled with anti-laminin α2 (LAMA2) and mesangial cells were localized with anti-integrin α8 (INTα8), demonstrating co-localization of laminin 211 with mesangial cell extension in capillary loops. Images D-F were taken with 40x1.3 n.a. oil; images G-I were taken with 63x1.4 n.a. oil with 2X zoom.

The α8 integrin has been shown to be strongly and exclusively expressed on the surface of mesangial cells of mice, rats, and humans.[34] As demonstrated in tissue from a WT dog, Fig 5A–5C, integrin α8 can be used as a mesangial cell marker in canine tissue as compared with the GBM marker α5 type IV collagen.

**Fig 6:**
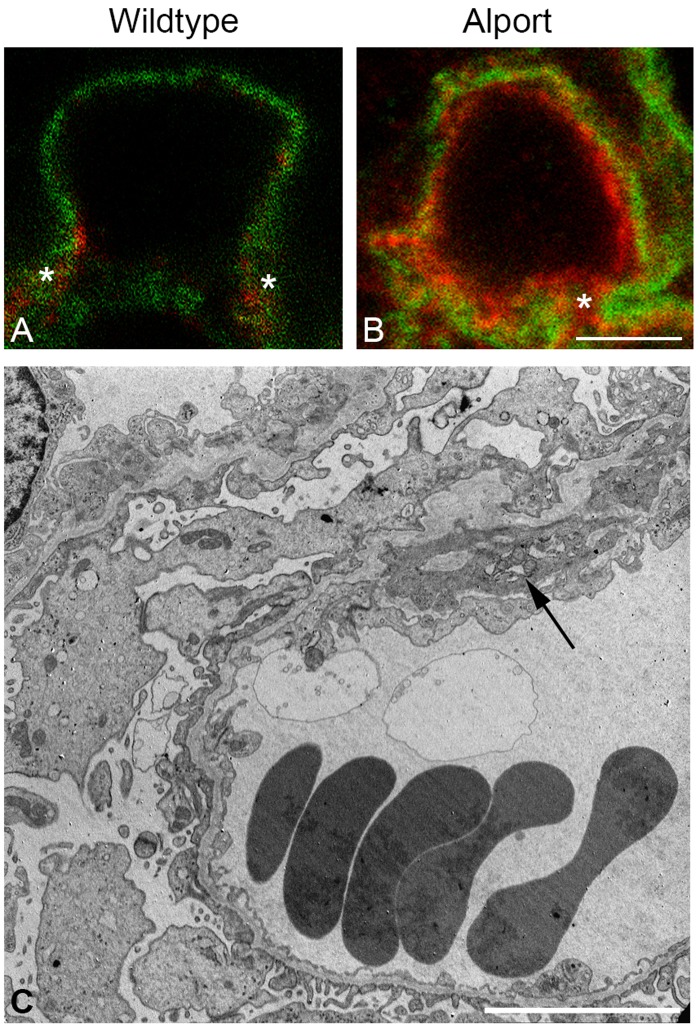
Mesangial cell process extension into the GBM of AS but not WT dogs. A-B: Dual immunofluorescence immunostaining of kidney from a WT dog and an AS dog, 63x1.4 n.a. oil with 3X zoom. Anti-laminin β2 and anti-integrin α8 antibodies were used to stain the GBM and mesangial cells, respectively. Staining reveals distinct delineation of mesangium absent from the GBM of the normal dog (A) but extension of mesangium within the GBM of the AS dog (B). C: Transmission electron microscopy of kidney tissue from an AS dog at milestone 2. Cytoplasmic extensions, also described as cellular interpositioning, are observed at the base of the capillary loops, consistent with invasion of mesangial cell processes (arrow) corresponding with extension of the mesangium (B).

Using dual immunofluorescence labeling with laminin α2 and integrin α8 in Alport mice, the source of GBM laminin 211 was shown to originate from mesangial cell processes that invade into capillary loops.[23] This same dual immunostaining was performed on canine kidney tissue. Fig 5D–5I shows intense co-localization of laminin α2 and integrin α8 outlining the capillary loop in an AS dog at MS 2, supporting that laminin α2 deposition is correlated with mesangial cells. Additionally, dual immunofluorescence staining was performed on kidney tissue from both a WT and AS dog using laminin β2 to stain the GBM with integrin α8 to stain the mesangium. Mesangial extension was clearly absent from the GBM in the WT dog while extension of mesangial cells within the GBM was observed in the AS dog (Fig 6A and 6B).

This corresponded with the TEM findings of mild cellular interpositioning (cytoplasmic extensions) along the capillary loops, which is consistent with invasion of mesangial cell processes (Fig 6C). This finding corresponded with an increased UPC in the AS dogs. Collectively, these data support that, as determined in the mouse, the unique deposition of laminin 211 within the GBM is likely a result of mesangial cell invasion of capillary loops in dogs with AS.

## Discussion

Mutations in α3, α4, or α5 type IV collagen genes result in absence of the normal type IV collagen composition of the GBM, permitting α1α1α2 type IV collagen to predominate. The thinner and less cross-linked composition of α1α1α2 type IV collagen likely allows for increased biomechanical strain in the capillary tuft due to increasing blood pressure as evidenced by acceleration of glomerular damage in salt-induced hypertensive mice.[35] This added stress on the capillary loop induces mesangial cell process invasion and contributes to initiation of disease.[23, 35, 36] The abnormal deposition of laminin 211 in the GBM is a feature that has been described as unique to AS.[21] As noted previously in mice and as seen in this study in dogs, the accumulation of 211 seems especially prominent in regions of the GBM that appear thickened on IF staining.[23] Using ferritin injections, these thickened areas have been shown to correlate with regions of loosely assembled or degraded extracellular matrix of the Alport GBM where permeability defects are present, and it is the deposition of aberrant laminins that contributes to these defects.[37] Using integrin α8 as a mesangial cell marker[34], we were able to show that, as reported in the mouse[23], there is extension of mesangial cell processes into the capillary loop of AS dogs and that the aberrant laminin 211 deposition in the GBM corresponds with these invading mesangial cell processes.

To further support the relationship between mesangial cell process invasion and deposition of laminin 211 in the GBM, mice with a deletion of CD151 have also been evaluated.[23] These mice have abnormalities of the adhesive interface between the podocyte pedicles and the GBM and display progressive morphological changes in the GBM similar to that in AS. Evaluation of glomeruli from these mice also demonstrates mesangial cell process invasion and GBM laminin 211 deposition supporting that, regardless of the cause of structural change, increased biomechanical strain on the capillary tuft stimulates mesangial cells to react. It is notable in this regard that CD151 null mice show accelerated progression of glomerular disease under conditions of hypertension, similar to Alport mice.[38] Additionally, evaluation of capillary tufts in glomeruli of integrin α1-null Alport mice (integrin α1 is important for mesangial cell expansion) have reduced mesangial process invasion and thus reduction of laminin 211, further supporting that laminin 211 originates from mesangial cells.[22] Immunogold-labeled integrin α8 is present in blebs noted in the subendothelial region of capillary loops in AS mice but not WT mice.[24] Recently, three-dimensional electron microscopy analysis of the glomerular structure of Alport mice identified mesangial cell processes invasion inside the GBM, along the mesangial aspect of the glomerular capillary loop.[39] In AS, mesangial cell invasion ultimately leads to an inflammatory response, likely in part driven by laminin 211-mediated FAK activation in podocytes, which is responsible for disease progression, including the development of glomerulosclerosis and tubulointerstitial fibrosis. While mesangial cell filopodia invasion has been shown to be mediated by biomechanical strain induced expression of endothelin-1 in endothelial cells, which in turn activates endothelin A receptors (ET_A_R) on mesangial cells leading to Rac1/CDC42 activation mechanism in mice [9, 23], further evaluation to explore this mechanism in the dog is needed.

In addition to demonstrating mesangial cell process invasion as an initiating event in dogs with AS, this study also allowed for comparison of clinical and structural changes throughout the course of disease through serial evaluations of individual dogs. XLAS is a hereditary progressive glomerular disease that typically results in rapidly progressive renal failure in affected males. Many affected individuals either do not have, or are not aware of, a family history of the disease and are not diagnosed until GFR declines, when clinical signs of disease become evident. In this study, comparison of serial biopsies with concurrent clinical data during the course of disease showed that significant pathologic changes to the kidney occur well before clinical markers of decreased GFR are altered. For instance, in AS dogs, sCr and SDMA did not show statistically significant changes until around milestone 3, while identification of fibronectin using IF evaluation of kidney tissue suggests instigation of fibrosis as early as milestone 1. On average, dogs in this study were around 28 weeks of age at MS 3 and all of the AS dogs evaluated in this study succumbed to disease before one year of age. Thus, approximately half of their lifespan was complete before disease was detectable by estimators of GFR. In contrast, microalbuminuria was highly sensitive to detection of structural changes evident with only electron microscopy and immunofluorescence staining. Institution of routine testing for microalbuminuria in human patients with hematuria and a family history of AS or renal failure without obvious cause may help ensure early clinical detection of AS.[40] From a clinical standpoint, early detection of proteinuria is paramount to early institution of therapy (e.g., ACE inhibition) that slows disease progression and helps extend life expectancy.[40, 41]

Currently, there are few accepted treatments for AS patients, none of which are directed at processes specific to initiation of disease. Understanding the pathogenesis of disease development helps determine the best targets for early intervention. In mice, FAK activation in podocytes occurs specifically where laminin 211 is being deposited, propagating disease progression.[24] It is conceivable that therapeutics that either inhibit FAK[24] or abate laminin 211 deposition could be developed for treatment of Alport syndrome. While mice have proven to be a useful model for understanding the molecular mechanisms of AS and are helpful in identifying therapeutic targets at earlier stages of disease, large animal models need to be established for drug trials. In general, the dog provides a transition platform between the pre-clinical testing of novel therapeutic drugs in mice and their use in humans. This is important from both a therapeutic efficacy and safety standpoint, as dogs have been shown to better mimic human disease in many conditions.[30, 42] For AS specifically, as mentioned above, it has been recently recommended that evaluation for proteinuria (particularly microalbuminuria) is crucial as an early identifier for the diagnosis of AS. It has been shown in the mouse model that proteinuria can vary between normal and affected mice, thus making proteinuria more insensitive for detecting glomerular disease.[43] Even amongst the different mouse models of AS themselves, onset of proteinuria can vary.[11, 14, 15, 44, 45] Therefore, one must be cognizant of these variations when choosing a mouse model for therapeutic study. Our results show that the distinction of WT versus AS dogs based on proteinuria is evident even at earlier stages of disease, making it a better model to monitor response to therapy. Additionally, the larger size of the dog along with the increased life span of AS dogs allows for serial evaluation of disease progression during therapeutic trials. These factors also allow for a better understanding of the long-term effects of new therapies. While AS itself only accounts for approximately 3% of end stage renal disease in children, the prevalence of CKD in the United States has risen dramatically.[46] Therefore, establishing a large animal model for CKD may be of broad importance for testing therapeutics. Given the rapidly progressive nature of AS in mice and dogs, AS serves as a good model for CKD development in general.

In summary, these findings collectively support, in a large animal model, the induction of mesangial cell filopodial invasion of the glomerular capillary tuft leading to the irregular deposition of mesangial laminin 211 in the GBM as an early initiating event in Alport glomerular pathology. Because of the similarities observed among canine and human disease progression, these findings also provide support that the dog is a suitable large animal model for evaluation of AS disease progression and novel therapeutic trials.

## Supporting Information

S1 Fig Controls for non-specific staining for immunofluorescence results presented in this paper. It is possible that cross reactivity of host serums in which the specific antibodies were raised might provide non-specific results. To control for this, we used the host serum in combination with the specific antibodies we employed in this work. The dual stains for which these apply are listed on the left side of the figure columns. NGS, normal goat serum; NMS, normal mouse serum; NRS, normal rabbit serum. Lamα2, laminin alpha 2 chain; Lamβ2; laminin beta 2 chain; Intα8, integrin alpha 8.
(TIFF)
