# Supplementary figures and images for: X-Linked Alport Dogs Demonstrate Mesangial Filopodial Invasion of the Capillary Tuft as an Early Event in Glomerular Damage
Source: PLoS One. 2016 Dec 13;11(12):e0168343. doi: 10.1371/journal.pone.0168343 (PMC5154607; doi:10.1371/journal.pone.0168343)

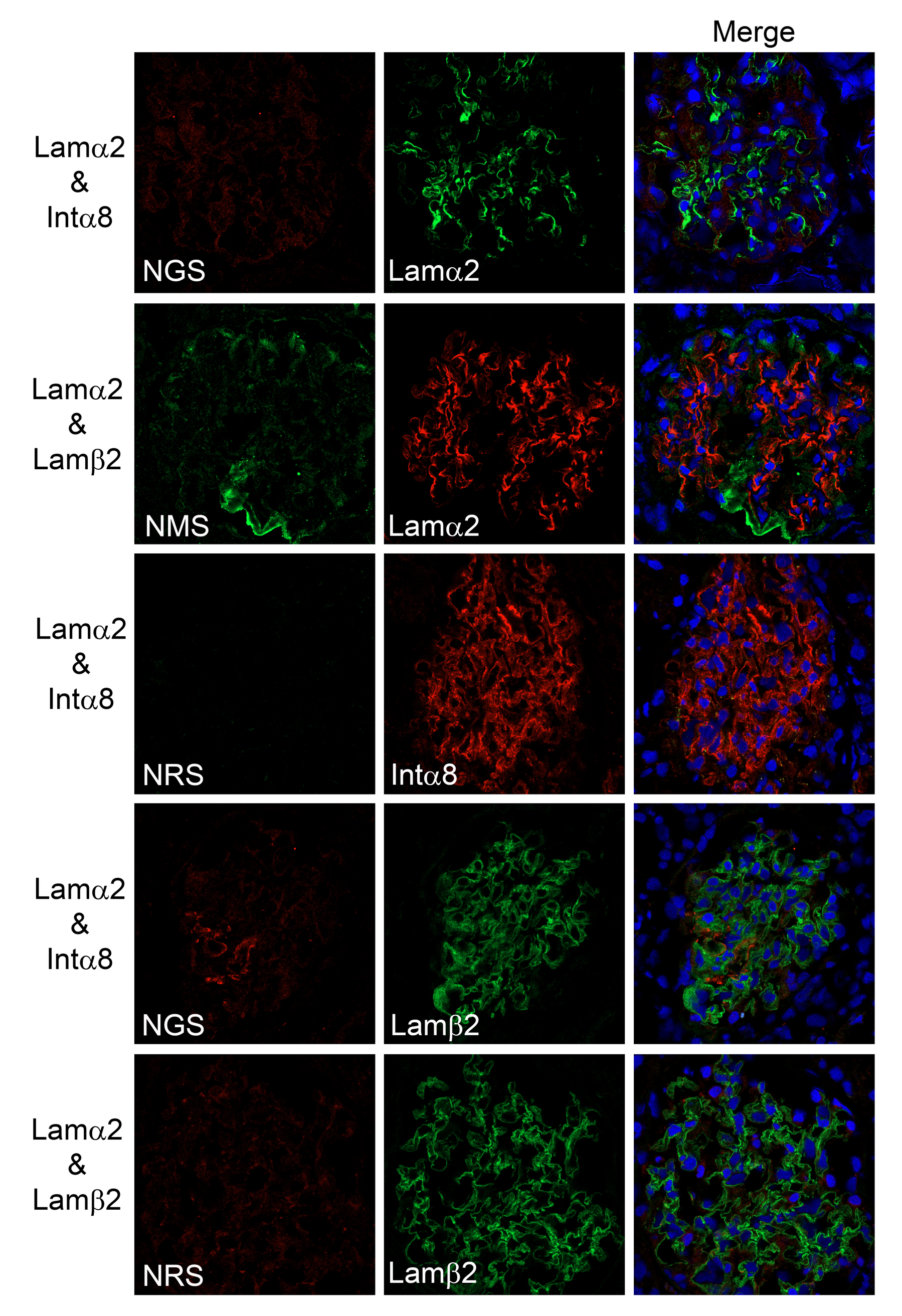

Supplement: S1 Fig — It is possible that cross reactivity of host serums in which the specific antibodies were raised might provide non-specific results. To control for this, we used the host serum in combination with the specific antibodies we employed in this work. The dual stains for which these apply are listed on the left side of the figure columns. NGS, normal goat serum; NMS, normal mouse serum; NRS, normal rabbit serum. Lamα2, laminin alpha 2 chain; Lamβ2; laminin beta 2 chain; Intα8, integrin alpha 8. (TIFF) [file pone.0168343.s001.tiff]
